# 5-Methyl-4-(5-methyl-3-oxo-2-phenyl-2,3-di­hydro-1*H*-pyrazol-4-yl)-2-phenyl-1*H*-pyrazol-3(2*H*)-one

**DOI:** 10.1107/S2414314620001212

**Published:** 2020-02-11

**Authors:** Gregory L. Powell, Brad A. Rix

**Affiliations:** a Department of Chemistry & Biochemistry, Abilene Christian University, Abilene, Texas 79699-8132, USA; Vienna University of Technology, Austria

**Keywords:** crystal structure, pyrazolone rings, hydrogen bonding

## Abstract

The title compound, C_20_H_18_N_4_O_2_, known as bis­pyrazolone, was crystallized from dimethyl sulfoxide. The structure has ortho­rhom­bic (*Pbca*) symmetry at 150 K, and displays both intra- and inter­molecular hydrogen bonding through C—H⋯O and N—H⋯O contacts, respectively. None of the phenyl and pyrazolone rings in the mol­ecule are coplanar. The dihedral angle between the pyrazolone rings is 66.18 (5)°.

## Abstract 2

The crystal structure of bis­pyrazolone, recrystallized from DMSO, indicates that the enamine tautomer is favored.

## Graphical abstract

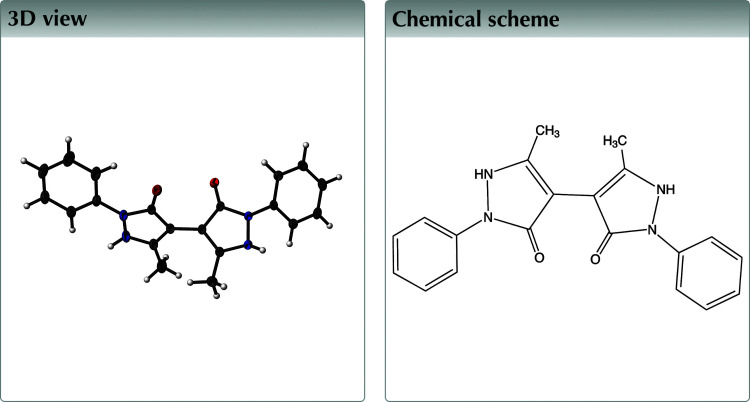

## Structure description

Pyrazolo­nes have been studied as anti­pyretics and analgesics (Brune, 1997 ▸; Badawey & El–Ashmawey, 1998 ▸; Gürsoy *et al.*, 2000 ▸), as anxiolytics (Geronikaki *et al.*, 2004 ▸), and as anti­hyperglycemic agents (Kees *et al.*, 1996 ▸). These compound types have also been found to have anti­oxidant and neuroprotective activities, and have been used to treat amyotrophic lateral sclerosis (ALS) and ischemia (Watanabe *et al.*, 2004 ▸; Yoshida *et al.*, 2006 ▸; Yuan *et al.*, 2008 ▸). Pyrazolo­nes have also been looked at as potential HIV-1 integrase inhibitors (Hadi, *et al.*, 2010 ▸). In addition to the multitude of possibilities in medicinal chemistry, pyrazolone research has led to prospective anti­microbial compounds (Chande *et al.*, 2007 ▸) and corrosion inhibitors (Elmorsi & Hassanein, 1999 ▸). The title compound, bis­pyrazolone, is primarily used as part of a pyridine-pyrazolone reagent for the detection of amine compounds. This method can qu­antify levels of cyanide (Epstein, 1947 ▸), ammonia and cyanate (Kruse & Mellon, 1952 ▸), and urea (Sharma *et al.*, 2013 ▸). It may also be used to determine the percentage of nitro­gen in steel samples (Lear & Mellon, 1957 ▸). Bispyrazolone and similar derivatives have also been examined as color developers (Bavley, 1946 ▸) and as lubricating oil thickeners (McGrath and Pellegrini, 1961 ▸) for high-temperature greases.

**Figure 1:**
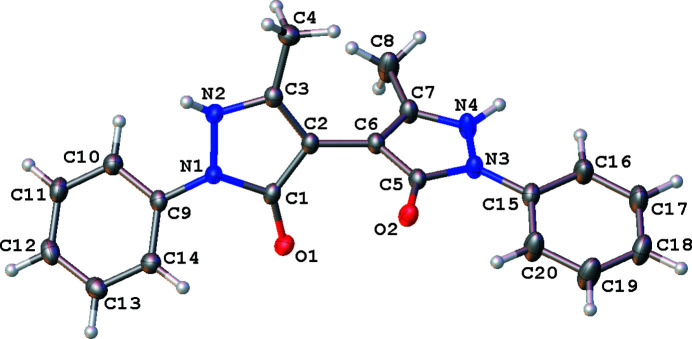
The title mol­ecule with the labeling scheme and displacement ellipsoids drawn at the 50% probability level.

**Figure 2:**
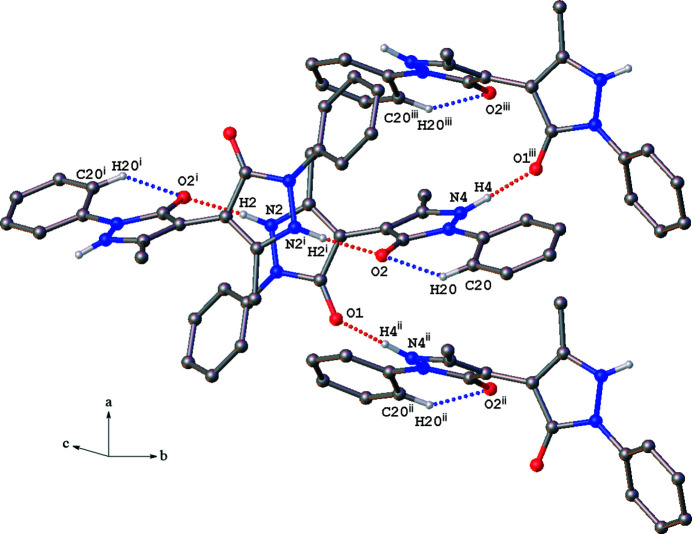
View of the intra­molecular (blue dotted lines) and inter­molecular (red dotted lines) hydrogen bond inter­actions. [Symmetry codes: (i) 1 − *x*, 1 − *y*, 1 − *z*; (ii) − 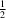 + *x*, 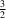 − *y*, 1 − *z*; (iii) 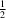 + *x*, 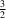 − *y*, 1 − *z*.]

In the crystal structure of the title compound, the mol­ecules are non-planar (Fig. 1 ▸). The dihedral angle between the two pyrazolone rings is 66.18 (5)°, while that between the phenyl rings is 39.44 (6)°. The ring systems in the halves of the mol­ecules have significantly different degrees of rotation with respect to one another. The dihedral angle between the C9–C14 phenyl ring and the N1/N2/C1–C3 pyrazolone ring is 34.29 (6)° while that between the C15–C20 phenyl ring and the N3/N4/C5–C7 pyrazolone ring is 13.75 (7)°. The latter is a consequence of intra­molecular C—H⋯O hydrogen bonding between the C20—H20 group on the phenyl ring and the O2 atom of the pyrazolone ring (Table 1 ▸, Fig. 2 ▸).

**Figure 3:**
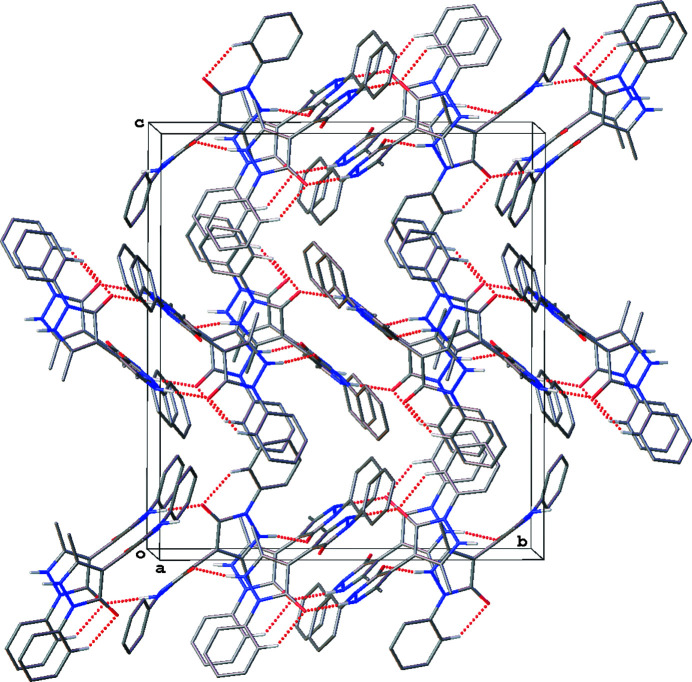
Packing of the mol­ecules viewed approximately along the *a* axis with hydrogen bonds shown as dotted lines.

In the crystal, the mol­ecules pack in a manner that maximizes inter­molecular hydrogen bonding. Both oxygen atoms and both N—H groups of each bis­pyrazolone mol­ecule are involved in forming four hydrogen bonds with three neighboring mol­ecules (Table 1 ▸, Fig. 2 ▸). The inter­molecular hydrogen bond axes lie approximately in the *bc* plane of the unit cell. Thus hydrogen-bonded sheets of the mol­ecules stack perpendicular to the *a* axis (Fig. 3 ▸).

## Synthesis and crystallization

A sample of the title compound was used as received from Sigma–Aldrich, and dissolved in hot di­methyl­sulfoxide. Colorless crystals were obtained by slow cooling of this solution to 298 K.

## Refinement

Crystal data, data collection and structure refinement details are summarized in Table 2 ▸.

## Supplementary Material

Crystal structure: contains datablock(s) I. DOI: 10.1107/S2414314620001212/wm4125sup1.cif

Structure factors: contains datablock(s) I. DOI: 10.1107/S2414314620001212/wm4125Isup2.hkl

Supporting information file. DOI: 10.1107/S2414314620001212/wm4125Isup3.cml

CCDC reference: 1949067

Additional supporting information:  crystallographic information; 3D view; checkCIF report

## Figures and Tables

**Table 1:** Hydrogen-bond geometry (Å, °)

*D*—H⋯*A*	*D*—H	H⋯*A*	*D*⋯*A*	*D*—H⋯*A*
N2—H2⋯O2^i^	0.89 (2)	1.85 (2)	2.7313 (14)	175.1 (18)
N4—H4⋯O1^ii^	0.93 (2)	1.81 (2)	2.7321 (15)	169.3 (18)
C20—H20⋯O2	0.95 (2)	2.23 (2)	2.8988 (17)	126.5 (16)

Symmetry codes: (i) 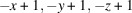 ; (ii) 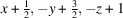 .

**Table 2:** Experimental details

Crystal data	
Chemical formula	C_20_H_18_N_4_O_2_
*M*_r_	346.38
Crystal system, space group	Orthorhombic, *P* *b* *c* *a*
Temperature (K)	150
*a*, *b*, *c* (Å)	8.7438 (1), 18.7561 (2), 20.8005 (2)
*V* (Å^3^)	3411.27 (6)
*Z*	8
Radiation type	Cu *K*α
μ (mm^−1^)	0.73
Crystal size (mm)	0.27 × 0.21 × 0.03

Data collection	
Diffractometer	Rigaku Oxford Diffraction SuperNova, Cu, AtlasS2 CCD
Absorption correction	Multi-scan (*CrysAlis PRO*; Rigaku OD, 2019 ▸)
*T*_min_, *T*_max_	0.874, 1.000
No. of measured, independent and observed [*I* > 2σ(*I*)] reflections	16964, 3336, 3020
*R*_int_	0.028
(sin θ/λ)_max_ (Å^−1^)	0.619

Refinement	
*R*[*F* ^2^ > 2σ(*F* ^2^)], *wR*(*F* ^2^), *S*	0.042, 0.113, 1.04
No. of reflections	3336
No. of parameters	307
H-atom treatment	All H-atom parameters refined
Δρ_max_, Δρ_min_ (e Å^−3^)	0.26, −0.25

Computer programs: *CrysAlis PRO* (Rigaku OD, 2019 ▸), *SHELXT* (Sheldrick, 2015*a* ▸), *SHELXL* (Sheldrick, 2015*b* ▸) and *OLEX2* (Dolomanov *et al.*, 2009 ▸).

## full crystallographic data

### Crystal data

**Table:**

C_20_H_18_N_4_O_2_	*D*_x_ = 1.349 Mg m^−^^3^
*M**_r_* = 346.38	Cu *K*α radiation, λ = 1.54184 Å
Orthorhombic, *P**b**c**a*	Cell parameters from 9599 reflections
*a* = 8.7438 (1) Å	θ = 4.7–72.3°
*b* = 18.7561 (2) Å	µ = 0.73 mm^−^^1^
*c* = 20.8005 (2) Å	*T* = 150 K
*V* = 3411.27 (6) Å^3^	Plate, clear colourless
*Z* = 8	0.27 × 0.21 × 0.03 mm
*F*(000) = 1456	

### Data collection

**Table:**

Rigaku Oxford Diffraction SuperNova, Cu, AtlasS2 CCD diffractometer	3336 independent reflections
Radiation source: micro-focus sealed X-ray tube, SuperNova (Cu) X-ray Source	3020 reflections with *I* > 2σ(*I*)
Mirror monochromator	*R*_int_ = 0.028
Detector resolution: 5.2387 pixels mm^-1^	θ_max_ = 72.6°, θ_min_ = 4.3°
ω scans	*h* = −7→10
Absorption correction: multi-scan (*CrysAlis PRO*; Rigaku OD, 2019)	*k* = −22→23
*T*_min_ = 0.874, *T*_max_ = 1.000	*l* = −25→24
16964 measured reflections	

### Refinement

**Table:**

Refinement on *F*^2^	Primary atom site location: dual
Least-squares matrix: full	Hydrogen site location: difference Fourier map
*R*[*F*^2^ > 2σ(*F*^2^)] = 0.042	All H-atom parameters refined
*wR*(*F*^2^) = 0.113	*w* = 1/[σ^2^(*F*_o_^2^) + (0.0692*P*)^2^ + 1.0751*P*] where *P* = (*F*_o_^2^ + 2*F*_c_^2^)/3
*S* = 1.04	(Δ/σ)_max_ = 0.001
3336 reflections	Δρ_max_ = 0.26 e Å^−^^3^
307 parameters	Δρ_min_ = −0.25 e Å^−^^3^
0 restraints	

### Special details

**Table:**

Geometry. All esds (except the esd in the dihedral angle between two l.s. planes) are estimated using the full covariance matrix. The cell esds are taken into account individually in the estimation of esds in distances, angles and torsion angles; correlations between esds in cell parameters are only used when they are defined by crystal symmetry. An approximate (isotropic) treatment of cell esds is used for estimating esds involving l.s. planes.
Refinement. The H atoms bound to N2 and N4 were located in a difference map and refined. All hydrogen atoms were located in a difference map and were refined isotropically without constraints.

### Fractional atomic coordinates and isotropic or equivalent isotropic displacement parameters (Å^2^)

**Table:**

	*x*	*y*	*z*	*U*_iso_*/*U*_eq_	
O1	0.17850 (10)	0.59409 (5)	0.52406 (4)	0.0228 (2)	
O2	0.41464 (11)	0.62429 (5)	0.38211 (4)	0.0254 (2)	
N1	0.33303 (11)	0.51616 (5)	0.58208 (5)	0.0197 (2)	
N2	0.48738 (12)	0.51224 (6)	0.59817 (5)	0.0203 (2)	
H2	0.525 (2)	0.4688 (11)	0.6043 (9)	0.040 (5)*	
N3	0.49474 (13)	0.74084 (6)	0.40463 (5)	0.0228 (2)	
N4	0.53811 (15)	0.77703 (6)	0.45972 (6)	0.0283 (3)	
H4	0.587 (2)	0.8209 (12)	0.4598 (9)	0.043 (5)*	
C1	0.30864 (14)	0.57394 (6)	0.54173 (6)	0.0189 (3)	
C2	0.45779 (14)	0.60260 (6)	0.52877 (6)	0.0200 (3)	
C3	0.56144 (15)	0.56250 (6)	0.56271 (6)	0.0205 (3)	
C4	0.73088 (16)	0.56807 (8)	0.56622 (7)	0.0280 (3)	
H4A	0.760 (2)	0.5841 (11)	0.6082 (10)	0.047 (5)*	
H4B	0.769 (2)	0.6027 (12)	0.5330 (10)	0.048 (5)*	
H4C	0.781 (2)	0.5205 (13)	0.5591 (11)	0.057 (6)*	
C5	0.46008 (14)	0.67053 (6)	0.42150 (6)	0.0206 (3)	
C6	0.48650 (14)	0.66557 (6)	0.48898 (6)	0.0210 (3)	
C7	0.53634 (16)	0.73124 (7)	0.50942 (7)	0.0252 (3)	
C8	0.5806 (2)	0.75544 (8)	0.57491 (7)	0.0378 (4)	
H8A	0.615 (3)	0.7180 (16)	0.6015 (14)	0.088 (9)*	
H8B	0.657 (3)	0.7932 (13)	0.5731 (11)	0.060 (6)*	
H8C	0.490 (3)	0.7748 (16)	0.5982 (14)	0.086 (9)*	
C9	0.22459 (14)	0.47963 (6)	0.62063 (6)	0.0203 (3)	
C10	0.26090 (16)	0.46291 (7)	0.68395 (7)	0.0249 (3)	
H10	0.3582 (19)	0.4766 (9)	0.7016 (8)	0.028 (4)*	
C11	0.15554 (16)	0.42644 (7)	0.72142 (7)	0.0282 (3)	
H11	0.1800 (19)	0.4165 (9)	0.7650 (9)	0.030 (4)*	
C12	0.01470 (16)	0.40714 (7)	0.69603 (7)	0.0280 (3)	
H12	−0.060 (2)	0.3814 (9)	0.7228 (9)	0.033 (4)*	
C13	−0.02218 (15)	0.42573 (7)	0.63351 (7)	0.0264 (3)	
H13	−0.122 (2)	0.4129 (9)	0.6151 (8)	0.031 (4)*	
C14	0.08272 (15)	0.46155 (7)	0.59499 (7)	0.0234 (3)	
H14	0.0610 (18)	0.4727 (9)	0.5502 (8)	0.025 (4)*	
C15	0.47280 (15)	0.77740 (7)	0.34550 (6)	0.0232 (3)	
C16	0.52997 (19)	0.84583 (8)	0.33728 (8)	0.0349 (4)	
H16	0.585 (2)	0.8683 (12)	0.3736 (11)	0.056 (6)*	
C17	0.5073 (2)	0.88090 (8)	0.27926 (8)	0.0388 (4)	
H17	0.552 (2)	0.9301 (11)	0.2736 (10)	0.046 (5)*	
C18	0.4295 (2)	0.84888 (8)	0.22973 (7)	0.0368 (4)	
H18	0.417 (2)	0.8738 (10)	0.1885 (9)	0.040 (5)*	
C19	0.3694 (2)	0.78142 (9)	0.23859 (8)	0.0440 (4)	
H19	0.313 (2)	0.7578 (12)	0.2045 (11)	0.054 (6)*	
C20	0.3906 (2)	0.74557 (8)	0.29632 (7)	0.0367 (4)	
H20	0.352 (2)	0.6986 (12)	0.3029 (10)	0.051 (5)*	

### Atomic displacement parameters (Å^2^)

**Table:**

	*U*^11^	*U*^22^	*U*^33^	*U*^12^	*U*^13^	*U*^23^
O1	0.0240 (5)	0.0187 (4)	0.0256 (5)	0.0027 (3)	−0.0025 (4)	0.0029 (3)
O2	0.0357 (5)	0.0157 (4)	0.0249 (5)	−0.0017 (3)	−0.0066 (4)	0.0006 (3)
N1	0.0195 (5)	0.0171 (5)	0.0225 (5)	0.0002 (4)	−0.0011 (4)	0.0039 (4)
N2	0.0209 (5)	0.0164 (5)	0.0236 (6)	0.0011 (4)	−0.0016 (4)	0.0029 (4)
N3	0.0324 (6)	0.0162 (5)	0.0197 (5)	−0.0033 (4)	−0.0008 (4)	0.0012 (4)
N4	0.0449 (7)	0.0185 (5)	0.0213 (6)	−0.0102 (5)	−0.0016 (5)	0.0012 (4)
C1	0.0256 (6)	0.0143 (5)	0.0167 (6)	0.0014 (4)	−0.0002 (5)	0.0000 (4)
C2	0.0255 (6)	0.0156 (6)	0.0190 (6)	−0.0012 (4)	−0.0001 (5)	0.0005 (5)
C3	0.0248 (6)	0.0167 (5)	0.0200 (6)	−0.0008 (5)	0.0007 (5)	−0.0007 (5)
C4	0.0241 (7)	0.0275 (7)	0.0324 (8)	−0.0017 (5)	−0.0014 (6)	0.0024 (6)
C5	0.0231 (6)	0.0147 (5)	0.0241 (6)	0.0002 (4)	0.0000 (5)	0.0022 (5)
C6	0.0246 (6)	0.0166 (6)	0.0219 (6)	−0.0014 (5)	0.0006 (5)	0.0018 (5)
C7	0.0339 (7)	0.0193 (6)	0.0224 (7)	−0.0040 (5)	0.0005 (5)	0.0014 (5)
C8	0.0647 (11)	0.0249 (7)	0.0238 (7)	−0.0129 (7)	−0.0052 (7)	−0.0002 (6)
C9	0.0236 (6)	0.0141 (5)	0.0233 (6)	0.0005 (4)	0.0021 (5)	0.0019 (4)
C10	0.0266 (6)	0.0227 (6)	0.0255 (7)	−0.0013 (5)	−0.0012 (5)	0.0033 (5)
C11	0.0332 (7)	0.0262 (6)	0.0253 (7)	0.0020 (5)	0.0031 (6)	0.0079 (5)
C12	0.0275 (7)	0.0216 (6)	0.0350 (8)	0.0008 (5)	0.0096 (6)	0.0051 (5)
C13	0.0229 (6)	0.0206 (6)	0.0357 (8)	−0.0004 (5)	0.0013 (6)	0.0010 (5)
C14	0.0245 (6)	0.0198 (6)	0.0258 (7)	0.0009 (5)	−0.0009 (5)	0.0012 (5)
C15	0.0285 (6)	0.0192 (6)	0.0218 (6)	0.0020 (5)	0.0035 (5)	0.0035 (5)
C16	0.0443 (8)	0.0274 (7)	0.0330 (8)	−0.0110 (6)	−0.0079 (7)	0.0083 (6)
C17	0.0509 (9)	0.0276 (7)	0.0381 (9)	−0.0095 (6)	−0.0046 (7)	0.0127 (6)
C18	0.0549 (9)	0.0295 (7)	0.0261 (7)	0.0019 (7)	−0.0006 (7)	0.0088 (6)
C19	0.0753 (12)	0.0302 (7)	0.0265 (8)	−0.0055 (8)	−0.0128 (8)	0.0028 (6)
C20	0.0616 (10)	0.0210 (7)	0.0274 (8)	−0.0076 (7)	−0.0076 (7)	0.0029 (6)

### Geometric parameters (Å, º)

**Table:**

O1—C1	1.2540 (15)	C8—H8C	1.00 (3)
O2—C5	1.2577 (16)	C9—C10	1.3908 (18)
N1—N2	1.3924 (15)	C9—C14	1.3922 (18)
N1—C1	1.3873 (15)	C10—H10	0.962 (17)
N1—C9	1.4182 (16)	C10—C11	1.3870 (19)
N2—H2	0.89 (2)	C11—H11	0.950 (18)
N2—C3	1.3608 (16)	C11—C12	1.388 (2)
N3—N4	1.3848 (15)	C12—H12	0.986 (18)
N3—C5	1.3979 (15)	C12—C13	1.384 (2)
N3—C15	1.4211 (16)	C13—H13	0.982 (18)
N4—H4	0.93 (2)	C13—C14	1.3908 (19)
N4—C7	1.3442 (17)	C14—H14	0.973 (17)
C1—C2	1.4361 (17)	C15—C16	1.3880 (19)
C2—C3	1.3732 (18)	C15—C20	1.385 (2)
C2—C6	1.4638 (17)	C16—H16	0.99 (2)
C3—C4	1.4870 (18)	C16—C17	1.389 (2)
C4—H4A	0.96 (2)	C17—H17	1.01 (2)
C4—H4B	1.01 (2)	C17—C18	1.373 (2)
C4—H4C	1.00 (2)	C18—H18	0.984 (19)
C5—C6	1.4255 (18)	C18—C19	1.382 (2)
C6—C7	1.3739 (18)	C19—H19	0.97 (2)
C7—C8	1.487 (2)	C19—C20	1.389 (2)
C8—H8A	0.94 (3)	C20—H20	0.95 (2)
C8—H8B	0.98 (2)		
			
N2—N1—C9	119.13 (10)	H8A—C8—H8B	110 (2)
C1—N1—N2	109.61 (10)	H8A—C8—H8C	104 (2)
C1—N1—C9	128.07 (10)	H8B—C8—H8C	108 (2)
N1—N2—H2	116.1 (12)	C10—C9—N1	119.46 (11)
C3—N2—N1	107.13 (10)	C10—C9—C14	120.74 (12)
C3—N2—H2	122.7 (12)	C14—C9—N1	119.80 (12)
N4—N3—C5	108.30 (10)	C9—C10—H10	120.3 (10)
N4—N3—C15	121.11 (10)	C11—C10—C9	119.46 (13)
C5—N3—C15	130.03 (11)	C11—C10—H10	120.3 (10)
N3—N4—H4	124.2 (12)	C10—C11—H11	118.9 (10)
C7—N4—N3	108.67 (11)	C10—C11—C12	120.27 (13)
C7—N4—H4	124.8 (12)	C12—C11—H11	120.8 (10)
O1—C1—N1	123.52 (11)	C11—C12—H12	120.1 (10)
O1—C1—C2	131.01 (11)	C13—C12—C11	119.90 (13)
N1—C1—C2	105.45 (10)	C13—C12—H12	120.0 (10)
C1—C2—C6	124.33 (11)	C12—C13—H13	120.7 (10)
C3—C2—C1	107.32 (11)	C12—C13—C14	120.61 (13)
C3—C2—C6	128.27 (12)	C14—C13—H13	118.7 (10)
N2—C3—C2	110.12 (11)	C9—C14—H14	119.2 (10)
N2—C3—C4	119.75 (11)	C13—C14—C9	118.99 (12)
C2—C3—C4	130.11 (12)	C13—C14—H14	121.7 (10)
C3—C4—H4A	109.2 (12)	C16—C15—N3	120.27 (13)
C3—C4—H4B	110.2 (12)	C20—C15—N3	120.08 (12)
C3—C4—H4C	111.4 (13)	C20—C15—C16	119.63 (13)
H4A—C4—H4B	109.6 (17)	C15—C16—H16	118.3 (13)
H4A—C4—H4C	107.4 (18)	C15—C16—C17	119.58 (14)
H4B—C4—H4C	109.1 (17)	C17—C16—H16	122.1 (13)
O2—C5—N3	123.75 (12)	C16—C17—H17	118.7 (11)
O2—C5—C6	130.37 (11)	C18—C17—C16	121.05 (14)
N3—C5—C6	105.88 (10)	C18—C17—H17	120.2 (11)
C5—C6—C2	125.56 (11)	C17—C18—H18	120.2 (11)
C7—C6—C2	127.07 (12)	C17—C18—C19	119.25 (14)
C7—C6—C5	107.31 (11)	C19—C18—H18	120.6 (11)
N4—C7—C6	109.78 (12)	C18—C19—H19	120.9 (13)
N4—C7—C8	120.43 (12)	C18—C19—C20	120.51 (15)
C6—C7—C8	129.77 (12)	C20—C19—H19	118.6 (13)
C7—C8—H8A	113.1 (18)	C15—C20—C19	119.94 (14)
C7—C8—H8B	111.4 (14)	C15—C20—H20	118.5 (13)
C7—C8—H8C	110.3 (16)	C19—C20—H20	121.5 (13)
			
O1—C1—C2—C3	176.71 (13)	C1—C2—C6—C7	110.02 (16)
O1—C1—C2—C6	−0.3 (2)	C2—C6—C7—N4	−175.33 (13)
O2—C5—C6—C2	−3.0 (2)	C2—C6—C7—C8	3.0 (3)
O2—C5—C6—C7	179.71 (14)	C3—C2—C6—C5	116.89 (15)
N1—N2—C3—C2	5.32 (14)	C3—C2—C6—C7	−66.4 (2)
N1—N2—C3—C4	−175.97 (11)	C5—N3—N4—C7	1.98 (15)
N1—C1—C2—C3	−1.56 (14)	C5—N3—C15—C16	−173.56 (14)
N1—C1—C2—C6	−178.61 (11)	C5—N3—C15—C20	8.1 (2)
N1—C9—C10—C11	−179.24 (12)	C5—C6—C7—N4	1.88 (16)
N1—C9—C14—C13	180.00 (11)	C5—C6—C7—C8	−179.78 (16)
N2—N1—C1—O1	−173.60 (11)	C6—C2—C3—N2	174.55 (12)
N2—N1—C1—C2	4.83 (13)	C6—C2—C3—C4	−4.0 (2)
N2—N1—C9—C10	23.01 (16)	C9—N1—N2—C3	−167.76 (11)
N2—N1—C9—C14	−157.62 (11)	C9—N1—C1—O1	−14.3 (2)
N3—N4—C7—C6	−2.41 (16)	C9—N1—C1—C2	164.12 (12)
N3—N4—C7—C8	179.07 (14)	C9—C10—C11—C12	−0.3 (2)
N3—C5—C6—C2	176.64 (12)	C10—C9—C14—C13	−0.64 (19)
N3—C5—C6—C7	−0.62 (14)	C10—C11—C12—C13	−1.4 (2)
N3—C15—C16—C17	−179.85 (14)	C11—C12—C13—C14	2.2 (2)
N3—C15—C20—C19	−179.98 (15)	C12—C13—C14—C9	−1.17 (19)
N4—N3—C5—O2	178.89 (12)	C14—C9—C10—C11	1.40 (19)
N4—N3—C5—C6	−0.81 (14)	C15—N3—N4—C7	174.20 (12)
N4—N3—C15—C16	16.11 (19)	C15—N3—C5—O2	7.6 (2)
N4—N3—C15—C20	−162.19 (14)	C15—N3—C5—C6	−172.10 (12)
C1—N1—N2—C3	−6.34 (13)	C15—C16—C17—C18	−0.2 (3)
C1—N1—C9—C10	−134.57 (13)	C16—C15—C20—C19	1.7 (3)
C1—N1—C9—C14	44.80 (18)	C16—C17—C18—C19	1.8 (3)
C1—C2—C3—N2	−2.35 (14)	C17—C18—C19—C20	−1.6 (3)
C1—C2—C3—C4	179.11 (13)	C18—C19—C20—C15	−0.1 (3)
C1—C2—C6—C5	−66.70 (18)	C20—C15—C16—C17	−1.5 (2)

### Hydrogen-bond geometry (Å, º)

**Table:**

*D*—H···*A*	*D*—H	H···*A*	*D*···*A*	*D*—H···*A*
N2—H2···O2^i^	0.89 (2)	1.85 (2)	2.7313 (14)	175.1 (18)
N4—H4···O1^ii^	0.93 (2)	1.81 (2)	2.7321 (15)	169.3 (18)
C20—H20···O2	0.95 (2)	2.23 (2)	2.8988 (17)	126.5 (16)

Symmetry codes: (i) −*x*+1, −*y*+1, −*z*+1; (ii) *x*+1/2, −*y*+3/2, −*z*+1.
